# Counting what counts: Time for an annual statistical update on neurologic health equity

**DOI:** 10.1016/j.neuros.2026.100071

**Published:** 2026-09-13

**Authors:** Bruce Ovbiagele

**Affiliations:** Department of Neurology, University of California, San Francisco, United States of America

**Keywords:** Neurological, Disparities, Epidemiology, Statistics, Report, Equity, United States

## Abstract

**Background:**

Neurologic diseases are among the leading causes of death and disability in the United States, yet the magnitude, distribution, and trajectory of neurologic disparities remain difficult to monitor across diseases and populations.

**Opportunity:**

Other fields have established powerful recurring statistical resources that have become essential references for clinicians, researchers, policymakers, and the public. For instance, the American Heart Association has produced an annual *Heart Disease and Stroke Statistics Update* for decades, integrating contemporary data on disease burden, risk factors, outcomes, disparities, and economic costs. Comparable models exist for cancer (American Cancer Society Cancer Statistics) and kidney disease (US Renal Data System Annual Data Report). These models demonstrate the value of a recognizable, authoritative statistical report that is updated as new evidence emerges.

**Proposal:**

This Viewpoint proposes an annual U.S. Neurologic Equity Annual Report (NEAR), modeled directly on these precedents, outlines its scope, candidate federal and private data sources, and strategic partnerships. NEAR would synthesize the most recent nationally representative data on incidence, prevalence, mortality, disability-adjusted life years, risk factors, health-care utilization, access and quality of care, and economic costs. Whenever data permit, estimates will be stratified by race/ethnicity, sex/gender, age, and socioeconomic characteristics.

**Conclusion:**

Neurology needs a recurring statistical framework that makes inequities measurable, comparable, and visible from year to year. NEAR would provide a common evidence base for identifying populations at greatest risk, tracking whether disparities are widening or narrowing, prioritizing research and prevention, guiding equitable allocation of resources, and evaluating progress toward neurologic health equity.

## Introduction

Neurologic diseases are now among the leading causes of death and disability in the United States, and the burden falls unevenly across populations defined by race and ethnicity, sex, geography, and socioeconomic position.^1^ Yet our ability to properly characterize and routinely track these differences across neurological disease entities, nationally, remains remarkably fragmented.^2,3^ For instance, i) a clinician can find statistics about stroke from one source, Alzheimer’s disease from another, Parkinson’s disease from a third, epilepsy from a fourth, and multiple sclerosis from a fifth; ii) a researcher seeking national mortality statistics may consult the National Center for Health Statistics, while another researcher studying disability-adjusted life year (DALYs) may turn to the Global Burden of Disease study; iii) a policymaker examining economic costs may encounter estimates generated using entirely different methodologies; and iv) a community organization seeking information about disparities may find data that are several years old, use different racial and ethnic classifications, or lack geographic granularity. The result is an epidemiologic paradox, i.e., the field of neurology possesses enormous amounts of data but lacks a common annual statistical narrative about neurologic disparities.^3^ This gap is consequential. Without a recurring reference point, it becomes a) difficult to determine whether disparities are improving, worsening, or remaining unchanged; b) difficult to compare the relative burden of different neurologic diseases across populations; c) difficult to determine whether improvements in overall neurologic outcomes are being shared equitably; and d) difficult for policymakers to recognize neurologic health inequity as a measurable national priority.

Closing this gap requires more than another single-disease epidemiologic report. What is proposed here, and described in detail below, is a standing infrastructure distinguished from prior efforts, by four features taken together: (1) Annual updates with a fixed publication cadence, creating institutional memory and year-over-year comparability; (2) A cross-disease approach that synthesizes data across major neurologic conditions (stroke, dementia, Parkinson disease, epilepsy, migraine, multiple sclerosis, ALS, and others) using harmonized methods, enabling direct comparison of burden and inequity; (3) A disparities-first framework in which race/ethnicity, sex, socioeconomic position, and geography are primary stratifiers embedded in every core table rather than supplemental analyses; and (4) A standardized methodology with transparent disease definitions, age-standardization, data provenance, and limitations reporting, modeled on durable precedents in cardiology, oncology, and nephrology. By placing mortality, morbidity, disability, care access and quality, risk factors, and economic burden within the same annual framework, NEAR would make it possible to ask not only who is most affected, but whether inequities are widening, narrowing, or remaining stable and whether apparent changes are robust to differences in ascertainment and data source.

In 2023, following the Health Disparities and Inequities in Neurological Disorders (HEADWAY) workshop, the National Institute of Neurological Disorders and Stroke (NINDS) published a series of articles summarizing a multiyear health equity strategic planning process.^4^ In one of these articles, Woolf and colleagues examined neurologic mortality in the United States (U.S.) from 2010 through 2019 and found that overall age-adjusted neurologic mortality began increasing after 2013.^5^ Furthermore, Non-Hispanic Black populations consistently experienced higher neurologic mortality than non-Hispanic White, American Indian and Alaska Native, Hispanic, and non-Hispanic Asian populations. The disparity widened during the study period to the extent that the difference in neurologic mortality between non-Hispanic Black and non-Hispanic White populations increased from 4.2 deaths per 100,000 in 2011 to 7.0 per 100,000 in 2019.^5^ Most strikingly, the investigators estimated that 29,986 deaths would have been averted among non-Hispanic Black Americans during 2010–2019 if they had experienced the neurologic mortality rates of non-Hispanic White Americans.^5^ The corresponding estimates were even larger when Hispanic or non-Hispanic Asian mortality rates were used as the reference.

The analysis by Woolf et al. is not simply an epidemiologic observation. It is a measure of lost lives associated with inequity. The study also demonstrated that disparities are not limited to race and ethnicity. Higher neurologic mortality was observed among older adults, men, and adults older than 25 years without a high school diploma.^5^ Importantly, the authors emphasized that mortality captures only part of the neurologic burden. Nonfatal neurologic disease, disability, cognitive impairment, functional limitation, and caregiver burden remain incompletely characterized. That observation should be a call to action. If neurologic mortality disparities alone are associated with tens of thousands of potentially avertable deaths, what is the full burden when incidence, prevalence, disability, DALYs, quality of life, health-care access, and economic consequences are considered? However, we currently do not have an annual national instrument designed to answer that question.

The NINDS HEADWAY initiative was, by design, a one-time undertaking tied to a specific strategic planning cycle.^4^ It examined mortality in a single decade and focused primarily on a single comparator pair (non-Hispanic Black versus non-Hispanic White Americans), and it has not been repeated. Meanwhile, national mortality rates, hospitalization rates, and survey data documenting neurologic disparities are recompiled every year by federal agencies, yet are largely collectively synthesized, interpreted, and published, and publicized. To routinely inform researchers, clinicians, policy makers, and the public, neurologic health equity, like cardiovascular health, cancer, kidney disease, and diabetes, deserves a standing, annually updated, comprehensively sourced statistical report. This viewpoint article outlines the case for such a report, situates it among comparable efforts already sustained by other disease domains, proposes its scope and candidate data infrastructure, and describes the partnerships needed to sustain it.

## Current general state of affairs

Beyond the already mentioned one-time HEADWAY-associated neurologic mortality analysis,^5^ a few U.S. advocacy organizations have issued neurologic disease-specific reports on inconsistent schedules for individual conditions (e.g., Parkinson’s disease, multiple sclerosis).^6,7^ This fragmentation of neurological disease statistical reports in the U.S. has three consequences. First, cross-condition comparison is difficult, i.e., a clinician or policymaker cannot easily determine whether disparities in stroke mortality are narrowing while disparities in dementia prevalence are widening, because the underlying reports use different years, different comparator groups, and different data sources. Second, trend detection is weak, i.e., a single decade-long retrospective analysis, however rigorous, cannot distinguish a stable disparity from one that is actively widening or narrowing in response to policy or clinical interventions. Third, the absence of a recurring, authoritative product leaves a vacuum that is sometimes filled by inconsistent or outdated secondary citations in the literature and popular press. An annual update, built on a fixed and transparent methodology and modeled on precedents that have already proven durable across five other disease domains, would address all three problems.

Moreover, it is crucial that a recurring national neurological disease statistics report deliberately move beyond mortality. Mortality is essential because death is an unambiguous outcome and because death certificate data provide valuable population-level information. But neurologic disease often produces its greatest societal consequences without immediately causing death. A person living for 20 years with multiple sclerosis, epilepsy, Parkinson disease, migraine, traumatic brain injury, or dementia may experience profound disability, reduced employment, caregiver dependence, and health-care utilization without appearing prominently in mortality statistics. Thus, the proposed report should characterize neurologic disparities across a multidimensional framework.

## Existing models worthy of consideration

Fortunately, a solution already exists elsewhere in medicine. Several major disease domains already treat annual or periodic national and global disease-statistics reporting as routine infrastructure rather than a special undertaking. The historical importance of this model should be emphasized. Annual statistics do more than summarize current trends. They create an institutional memory for a field, and can become much needed foundation for a field, not merely another publication. Moreover, year after year, an annual report creates a common vocabulary of key questions:

- What is the burden?
- Is it increasing or decreasing?
- Who is most affected?
- Which risk factors are changing?
- Which populations experience the worst outcomes?
- What does the disease cost?
- Where are the largest gaps?
- Are interventions working?

A compelling and perhaps analogous model precedent to consider is that of the American Heart Association (AHA)’s Heart Disease and Stroke Statistics Update.^8,9^ The AHA’s Heart Disease and Stroke Statistics Update in collaboration with the National Heart, Lung, and Blood Institute (NHLBI) has been published annually for decades, aggregating incidence, prevalence, mortality, cost, and risk-factor data across federal surveillance systems into a single citable reference used routinely by clinicians, policymakers, journalists, and grant reviewers.^8,9^ The 2026 update includes data on multiple major disease categories and provides dedicated fact sheets examining race, ethnicity, sex, age, and other population dimensions.^9^ The AHA describes the statistics program as a resource for clinicians, researchers, policymakers, health administrators, advocates, and the public.^8,9^

Two other periodic statistical reports cover aspects of neurology, i) the Alzheimer’s Association publishes an annual comprehensive statistical report titled Alzheimer’s Disease Facts and Figures, which has been released every year since 2007, and tracks national and state data on disease prevalence, mortality, caregiving, and health care costs, alongside a special focus section on a timely topic;^10^ ii) the World Stroke Organization (WSO) independently develops and periodically publishes (triennially) a Global Stroke Fact Sheet.^11^ The 2025 fact sheet provides a concise, authoritative synthesis of contemporary global stroke epidemiology. It incorporates Global Burden of Disease estimates and presents incidence, prevalence, mortality, and DALYs in a format that can be communicated to clinicians, policymakers, advocates, and the public.^11^ The strength of this model is not simply that it contains numbers. It creates a common and periodic narrative about the state of stroke.

Beyond neurological disease entities, oncology, nephrology, and endocrinology maintain comparable reports. The American Cancer Society’s Cancer Statistics report has appeared annually since 1951, making it the longest-running example of this model and its report provides contemporary cancer incidence, mortality, survival, risk-factor, and prevention information and includes estimates by cancer type, sex, state, and race and ethnicity.^12^ The US Renal Data System (USRDS), supported by the National Institute of Diabetes and Digestive and Kidney Diseases and the Centers for Medicare & Medicaid Services, publishes an Annual Data Report on chronic kidney disease and end-stage renal disease epidemiology.^13^ Globally, the International Diabetes Federation (IDF) publishes a periodically updated Diabetes Atlas providing global, regional, and national prevalence estimates and projections.^14^ Table 1 summarizes elements of these select disease statistical updates.

These models are instructive as much for the operational challenges they’ve had to overcome as for their successes. The AHA Heart Disease and Stroke Statistics Update, for example, has had to periodically revise case definitions and data sources as federal surveillance systems changed, requiring the writing group to flag discontinuities explicitly so that trend lines remain interpretable across editions.^8,9^ The American Cancer Society’s report has had to contend with reporting lags in national cancer registries, which it addresses by publishing modeled projections alongside observed data rather than waiting for complete ascertainment.^12^ The USRDS has long grappled with the limits of claims-based data, which capture the Medicare-insured kidney disease population well but incompletely represent younger, uninsured, or commercially insured patients.^13^ WSO and Alzheimer’s Association reports highlight the need for tiered products (technical report plus fact sheets/infographics) to reach policymakers and the public without oversimplifying uncertainty. Sustaining any of these reports year after year has required durable funding, staffing, source harmonization, and governance structures that outlast any single champion or funding cycle. Each of these lessons directly shaped the neurologic equity report framework proposed below, i.e., an explicit methods appendix and data-provenance table to flag discontinuities and modeled versus observed estimates (see Methodological transparency, below); deliberate use of multiple, complementary federal data sources rather than a single claims-based system to offset the coverage gaps of any one source; a standing secretariat for continuous monitoring; a named, rotating governance structure intended to give the report institutional durability independent of any one individual or sponsor (see Partnerships and Governance, below); standardized International Classification of Diseases, Tenth Revision, Clinical Modification (ICD-10-CM) and Research, Condition, and Disease Categorization (RCDC) mappings with change logs, small-population methods (suppression, pooling, Bayesian smoothing); a standing “data gaps as outcome” table; and tiered dissemination.

Currently, there is no periodically published statistical report in the U.S. that covers the full range of neurologic conditions across key epidemiological parameters, let alone specifically examines disparate populations in the country. There also does not seem to be a periodically published statistical report for any disease entity in the U.S. that covers key epidemiological parameters for disparate populations. Compared to a conventional health statistics update, a health disparities statistics update would require focusing on additional areas of emphasis as shown in Table 2.

## AHA heart and stroke statistics update as a template

Rather than building a new model, adopting and adapting one that has already been stress-tested for more than two decades and remains the closest existing template for a neurologic annual statistical report may be prudent. The AHA Heart Disease and Stroke Statistics Update is produced annually by the AHA Council on Epidemiology and Prevention Statistics Committee and Stroke Statistics Subcommittee, in conjunction with the National Heart, Lung, and Blood Institute within the National Institutes of Health, National Center for Health Statistics (NCHS) within the Centers for Disease Control and Prevention (CDC), and other federal agencies.^8,9^ Four structural features of that process are directly transferable to neurology and, explain its durability and credibility.

- **Continuous, not episodic, data monitoring.** The AHA writing group continuously monitors and evaluates federal and other data sources throughout the year, with a systematic literature review extending through the year before writing, rather than convening a single retrospective analysis and disbanding.^8,9^ This continuous-surveillance model is what allows the report to appear on a fixed annual schedule rather than as an occasional special issue. This same continuous-monitoring principle underlies the WSO Global Stroke Fact Sheet’s periodic updates and the USRDS Annual Data Report.^11,13^
- **A standing, rotating volunteer writing group.** The AHA writing group has a named leadership and disclosed conflicts of interest.^8,9^ Each edition is produced by a named chair and vice chair leading a standing subcommittee of volunteer clinicians and scientists, government liaisons, and dedicated AHA staff, all of whom complete a disclosure questionnaire specifying any relationship that could be perceived as a conflict of interest.^8,9^ Membership rotates across editions, which distributes institutional memory, prevents any single organization or advocacy interest from dominating the report, and gives each year’s report identifiable, publicly accountable authorship.
- **A fixed core with an expandable scope.** The report’s core chapters (mortality, prevalence, risk factors, cost, quality of care) have remained stable since its earliest editions, which preserves year-over-year comparability, while new chapters have been added as the science and data infrastructure matured, most notably a dedicated brain health chapter and expanded global data beginning with the 2024 edition.^8,9^ This shows that a statistics update can evolve in scope without sacrificing the trend continuity that is its central analytic value.
- **Explicit engagement with structural drivers of inequity**. The 2024 edition explicitly frames disparities in cardiovascular outcomes within the context of the social determinants of health, rather than presenting stratified statistics as neutral background.^9^

Table 3 maps each of the AHA Update features onto a potential annual neurologic health equity statistical update and suggests the neurology-specific partner or mechanism that would fill the corresponding role. Since NHLBI and NCHS already collaborate with the AHA writing group on stroke content,^8,9^ an annual neurologic health equity statistical update built along the same lines would not compete with the AHA report or the WSO Global Stroke Fact Sheet,^11^ but would complement both, i.e., those reports could remain the definitive sources for stroke as a cardiovascular and global public health condition, while annual neurologic health equity statistical update extends the same rigor and cadence to the non-stroke neurologic conditions neither report covers in comparable depth (dementia, Parkinson disease, epilepsy, migraine, multiple sclerosis, and ALS), and adds the multi-condition, disparities-first stratification structure that is currently absent from the neurology literature entirely.

## Proposal for annual stats update on neurologic health equity

I propose establishing the **N**eurologic **E**quity **A**nnual **R**eport (NEAR), a recurring peer-reviewed, professionally sponsored report providing current US data on incidence, prevalence, mortality, disability-adjusted life-years (DALYs), health care cost, and care patterns for major neurologic conditions, stratified consistently across sex, race and ethnicity, geography, and socioeconomic position. Table 4 outlines the proposed scope of NEAR. I suggest an annual rather than biannual cadence for the peer-reviewed scientific report, both to align with the precedent set by the AHA, American Cancer Society, and USRDS reports and to generate a denser trend series for signal detection, while producing biannual or triannual updates of lay-person facing dashboard content for non-technical audiences. An annual publication schedule is a cadence for reporting, not necessarily a cadence for re-assessing whether a disparity has meaningfully changed, i.e., each edition of the report should present rolling multiyear trend lines rather than year-over-year point comparisons, since single-year fluctuations in national survey and claims data are rarely sufficient to distinguish a genuine change from statistical noise. This distinction matters because disparities do not move only in one direction. In some minoritized populations in the U.S., certain neurologic and related health disparities have narrowed over time, even if not as quickly as warranted, and an update that emphasizes trend context, rather than only the current-year snapshot, would allow NEAR to credibly report that progress alongside the areas where gaps are stable or widening.

To avoid spurious interpretation of single-year fluctuations, NEAR should pre-specify use of 3-year moving averages for rare conditions and small strata, publish uncertainty intervals, and distinguish short-term variability from sustained trajectories. Both absolute and relative disparity measures should be reported because the two can move in different directions and answer different equity questions.^15^ When data permit, annual percent change or comparable slope estimates, segmented or joinpoint approaches, and multiyear regression models should be considered, with prespecified rules for interpreting changes in disparity. A lack of statistical significance should not automatically be described as evidence of no change, particularly for small populations; conversely, statistically detectable changes should be distinguished from changes large enough to be practically meaningful. A dedicated *“Are disparities widening, narrowing, or stable?”* figure/table in each edition should summarize trend direction by condition and population, addressing the need to celebrate progress where it exists while maintaining accountability where gaps persist or widen. Methodologic changes that could affect trends (ICD coding updates, new case definitions) should be flagged explicitly, as done by AHA and USRDS.

- **What Diseases Should Be Included?** A central strength of the proposed update would be its breadth. The report should not necessarily be limited to the largest neurologic diseases by mortality. It can include conditions that collectively account for substantial disability, health-care utilization, lost productivity, or caregiver burden. The precise list should evolve over time. Diseases with insufficient data for reliable annual estimates should not be excluded permanently; rather, they should be identified as data gaps. Indeed, the report should explicitly track data gaps as an outcome.
- **Risk-Factor Dimension.** Neurologic disparities cannot be understood solely by counting neurologic diagnoses. Many neurologic conditions are influenced by modifiable or potentially modifiable risk factors, including hypertension, diabetes, obesity, smoking, physical inactivity, diet, alcohol exposure, air pollution, environmental toxins, sleep disorders, social isolation, educational opportunity, and other social determinants. Stroke provides the clearest example. The 2025 World Stroke Organization *Global Stroke Fact Sheet* synthesized contemporary global data showing the enormous and increasing burden of stroke, including more than 160 million DALYs globally and substantial increases in incident strokes, stroke deaths, and DALYs between 1990 and 2021.^11^ The majority of global stroke deaths and DALYs occur in low-income and lower-middle-income countries. A neurological disparities report should connect risk-factor disparities to neurologic outcomes. Below are two examples of pathways an annual neurologic health equity surveillance should make visible.
  √ Hypertension → stroke → disability → loss of employment → caregiver burden → economic cost
  √ Social disadvantage → reduced access to preventive care → delayed diagnosis → more severe disease → increased disability → higher long-term cost
- **Economic Case.** Economic burden should be a core component rather than an optional appendix. Neurologic disease affects the economy through multiple pathways including direct medical expenditures; hospitalizations and emergency care; outpatient and specialty care; prescription medications; rehabilitation; long-term care; disability; absenteeism; reduced workforce participation; early retirement; unpaid caregiving; and premature mortality. The Alzheimer’s Association’s annual statistics report provides an instructive precedent.^10^ Its current report incorporates health and long-term care expenditures, unpaid caregiving, workforce effects, and other economic consequences into the annual assessment of dementia burden.^10^ A neurological disparities update could similarly ask not merely how many people disease have, but who bears the financial consequences of disease. This distinction is particularly important because direct economic costs can obscure inequity. A population with lower access to health care may have lower measured medical expenditures while simultaneously experiencing greater disability, lost productivity, and premature mortality. Consequently, economic analyses should distinguish health-care spending from total societal burden.
- **Race and Ethnicity.** Race and ethnicity should be central to the report, but the report should avoid treating racial categories as biological explanations for disparities. The Woolf et al. study provides a useful example.^5^ The authors documented substantial racial differences in neurologic mortality but situated those differences within broader historical, social, structural, and health-system contexts.^5^ A modern statistical update should therefore report race and ethnicity alongside factors such as educational attainment; household income; neighborhood deprivation; health insurance; rurality; geographic access to neurologists; transportation; language; immigration-related barriers where appropriate; environmental exposures; health-care quality; social isolation; disability; and other relevant social determinants. Not every statistic will support every stratification. Statistical stability and privacy must be preserved. However, the report should make explicit which dimensions were evaluated and which could not be measured.
- **Geography Matters.** Neurologic health is also geographically patterned. An annual update should provide maps demonstrating variation in incidence, mortality, disability, access to neurologists, treatment rates, and other outcomes across states and, when feasible, counties. Geographic data could reveal disparities that disappear in national averages. For example, two states may have identical national stroke mortality rates but very different patterns by race, rurality, poverty, and access to comprehensive stroke centers. The report should therefore incorporate a geographic equity dashboard.
- **Methodological transparency.** The report should publish a detailed methods appendix each year describing: disease definitions; International Classification of Diseases (ICD) coding; population denominators; age-standardization methods; race and ethnicity classification; handling of missing data; estimation methods; confidence or uncertainty intervals; suppression rules; economic-cost methodology; DALY methodology; changes from prior editions; and limitations. Where possible, estimates should be age-standardized to a consistent reference population so that comparisons across racial, ethnic, and geographic populations are not confounded by different age structures. When estimates are derived from different sources, the report should explicitly identify them rather than presenting them as interchangeable. A “data provenance” table should accompany every major statistic.
- **Candidate Data Infrastructure.** No single database can support the scope of NEAR; the report should ideally be a synthesis product drawing on multiple federal, quasi-federal, and private data assets that already exist and are refreshed on regular cycles, much as the AHA update draws on NCHS, NHLBI, and U.S. Centers for Medicare and Medicaid Services (CMS) sources rather than any single registry.^8,9^ Table 5 summarizes the potential principal candidate sources for the NEAR.

## Partnerships and governance

Sustaining an annual product requires a governance model that combines scientific independence with institutional durability. As detailed above, I propose adapting the AHA’s own governance structure directly: a named chair and vice chair, a rotating multi-stakeholder scientific steering committee with mandatory conflict-of-interest disclosure, and formal data-sharing and co-production relationships with federal partners, convened by a professional body with a brain health equity science mission such as Society for Equity Neuroscience (SEQUINS) in collaboration with a professional neurology society with an academic neurology mission such as the American Neurological Association (ANA). Supplementary Table 1 outlines candidate partners and their proposed roles. The NINDS Office of Global Health and Health Disparities, which oversaw the HEADWAY process, is a natural scientific and convening partner given its existing relationships with the data-generating federal agencies and its prior investment in this exact analytic question.^4^ Notably, NHLBI serves as federal co-producer of the AHA’s stroke statistics,^8,9^ so an annual NEAR update built on the same template would extend, rather than duplicate, an existing federal collaboration. Communities affected by disparities should have meaningful input into which outcomes are measured and how findings are interpreted.

## Dissemination and translation

A statistical update has value only if it reaches the audiences that can act on it. I propose a tiered dissemination model: (1) the peer-reviewed report itself, published in a neurology/clinical neuroscience journal; (2) a companion open-access policymaker and lay summary with data visualizations, comparable to the AHA’s own statistics-at-a-glance fact sheets; (3) a public, interactive online dashboard permitting users to query specific condition-by-stratum-by-year combinations; and (4) briefings to relevant congressional committees, NIH institute leadership, and state health departments timed to coincide with each annual release. Podcasts, webinars, and social-media summaries, modeled on formats already used by SEQUINS and the ANA, can extend reach to trainees and community stakeholders who will not read the primary report. See Supplementary Table 2 for an illustrative annual neurological disparities’ scorecard and Supplementary Table 3 for a proposed annual publication architecture.

Since a statistical update has value only if it reaches and is used by its intended audiences, NEAR’s governance group should track its own reach and influence as a matter of course, not as an afterthought. I would suggest a pre-specified evaluation framework for reach and influence, to be reported transparently in each edition’s appendix:

- **Academic uptake.** Annual citation count, Altmetric score, and tracking of NEAR inclusion in NIH grant applications (e.g., via NIH RePORTER keyword analysis), systematic reviews, and clinical guidelines.
- **Policy citation.** Number and type of policy documents referencing NEAR (e.g., Congressional testimony, state health department reports, Healthy People 2030 updates, U.S. Centers for Medicare & Medicaid Services equity reports, NINDS strategic plan progress reports).
- **Dashboard and digital engagement.** Unique users, query volume, geographic distribution, and return usage for the interactive dashboard; downloads of fact sheets; podcast/webinar attendance.
- **Media and public discourse.** Media mentions in lay press, specialty news (e.g., Neurology Today, Medscape), and social media impressions, analyzed by audience type.
- **Adoption by advocacy and community organizations.** Use of NEAR data in condition-specific organization reports (e.g., Alzheimer’s Association, Epilepsy Foundation), patient education materials, and community health needs assessments.
- **Equity of reach.** Assessment of whether NEAR data are being accessed and applied in high-disparity regions and by minority-serving institutions.

A logic model linking inputs → activities → outputs → outcomes → impact could be published with the first edition, and annual benchmarking against these indicators will allow iterative improvement and accountability. Reporting these indicators annually, alongside the statistical content itself, would let NEAR demonstrate its own value proposition using the same evidence-based approach it applies to neurologic disparities.

## Limitations and challenges

A foundational limitation deserves explicit mention. NEAR would rely on existing national data sources, and these sources do not count morbidity and mortality with equal validity across all populations. It needs to be acknowledged that reliance on existing national data sources, while economically feasible and necessary for sustainability, carries three inherent limitations that may disproportionately affect marginalized populations.

- Under-ascertainment due to structural barriers to care, i.e., individuals experiencing poverty, uninsurance, housing instability, or lack of transportation may delay or forgo medical presentation, leading to underestimation of true incidence, prevalence, and risk-factor burden in claims, discharge, and survey data. This is well-documented for neurologic conditions where access to specialty diagnosis (e.g., EEG for epilepsy, MRI for MS, cognitive testing for dementia) is required.^16,17^
- Differential coding and documentation quality by hospital resourcing, i.e., Black, Hispanic, and low-income patients disproportionately receive care at under-resourced safety-net hospitals with higher patient-to-coder ratios, less robust health information technology, and fewer neurology specialists to document specific diagnoses. Studies of hospital segregation have shown that Black and White Americans often receive care at different hospitals, and that hospitals serving higher proportions of Black patients have lower resources and performance on quality metrics.^18,19^ This can lead to systematic under-coding of neurologic conditions as secondary diagnoses, less specific ICD coding, and misclassification of cause of death on death certificates, biasing disparity estimates conservatively.
- Survey non-response and language barriers, i.e., national surveys have lower response rates among low-income, limited-English-proficiency, and institutionalized populations, and typically exclude incarcerated and unhoused persons. To address these concerns, NEAR should adopt a critical data appraisal framework in each edition: (a) report data completeness and missingness by stratum; (b) include a limitations box specifically discussing directionality of bias (e.g., likely underestimation of burden in socioeconomically disadvantaged groups); (c) where feasible, apply quantitative bias analysis and sensitivity analyses (e.g., comparing NVSS underlying vs. multiple cause coding, inpatient vs. outpatient vs. claims estimates); (d) triangulate across sources to assess consistency; and (e) maintain a “Data Equity Gaps” tracker listing conditions and strata for which current sources are inadequate and require targeted enhancement or primary data collection. This approach balances feasibility with scientific rigor and aligns with recommendations for health equity surveillance.^20,21^

Several additional challenges deserve explicit recognition. First, national epidemiologic data often lag the current year. The American Cancer Society explicitly addresses this challenge by producing projections when contemporary incidence and mortality data are not yet available.^10^ A neurological update could use a similar strategy, clearly distinguishing observed data from modeled estimates. Second, data-use agreements with U.S. Centers for Medicare & Medicaid Services (CMS) and some registries can take a year or more to establish and renew, which argues for securing multiyear agreements at program launch rather than annually. Third, small subgroup sizes, particularly for American Indian/Alaska Native populations who remain poorly represented in national datasets, and for rarer conditions such as amyotrophic lateral sclerosis, will require suppression rules, Bayesian small-area estimation, or multiyear pooling to protect both statistical reliability and participant privacy, with methods disclosed transparently in each release. Fourth, different studies use different disease definitions. The annual update should establish standardized definitions whenever possible. Fifth, coding and case-definition changes (e.g., ICD revisions, evolving dementia diagnostic criteria) can create artificial discontinuities in a multiyear trend series and must be flagged explicitly, as the AHA report does when its own case definitions change. Sixth, an observed disparity does not automatically establish its cause. The report should distinguish descriptive disparities from causal explanations. Seventh, race and ethnicity should be reported because they are essential dimensions of inequity, but findings should not be interpreted as evidence that racial categories themselves are biological causes of disease. Finally, sustained funding and editorial independence are essential: a report perceived as advocacy-driven rather than data-driven would lose the credibility that makes an annual statistics update useful to clinicians and policymakers in the first place. A named, rotating scientific steering committee with published conflict-of-interest disclosures, modeled on the AHA, American Cancer Society, and USRDS governance structures, can mitigate this risk.

## Conclusions

What gets measured consistently becomes visible, and what becomes visible can become a target for action. The evidence indicates that the need is urgent. The 2023 NINDS health equity strategic planning process demonstrated both the scientific feasibility and the substantial policy value of systematically quantifying neurologic health disparities in the United States. Converting the HEADWAY analysis into a recurring, annually updated, multi-condition statistics report is a logical extension of an operating model that other disease domains have already proven durable, built on data infrastructure that already exists and partnerships that are already in place.

An annual U.S. Neurological Disparities Statistics Update would not replace disease-specific research. It would amplify it. It would not replace government surveillance. It would translate and synthesize it. It would not simply report disparities. It would track them. Most importantly, it would establish a baseline against which progress could be measured. The objective should be ambitious but straightforward: Measure every major neurologic disparity, every year. Identify where inequities are greatest. Track whether they are improving or worsening and use those data to change the trajectory of neurologic health in the U.S. The neurology community has an opportunity to make disparities impossible to overlook. SEQUINS, NINDS, ANA, federal data agencies, and condition-specific advocacy organizations should convene a working group to formalize a U.S. Neurologic Equity Annual Report (NEAR), with a target of a first full edition within 2 to 3 years. The time has come to count neurologic health equity metrics, not once, but every year.

## Supplementary Material

Supplementary Tables 1-3

Supplementary material associated with this article can be found, in the online version, at https://doi.org/10.1016/j.neuros.2026.100071.

## Figures and Tables

**Table 1:** Established annual or recurring national and global disease-statistics reports produced by professional societies or international organizations.

Report	Sponsor	Journal / Venue	Disease Domain	Cadence
Heart Disease and Stroke Statistics Update^9^	American Heart Association, with NIH/NHLBI, NINDS, and CDC/NCHS	Circulation	Cardiovascular disease, including stroke	Annual
Global Stroke Fact Sheet^11^	World Stroke Organization	International Journal of Stroke	Stroke (global)	Periodic (approximately every 2–3 y)
Cancer Statistics^12^	American Cancer Society	CA: A Cancer Journal for Clinicians	Cancer (all sites)	Annual (since 1951)
USRDS Annual Data Report^13^	US Renal Data System (NIDDK and CMS)	American Journal of Kidney Diseases	Chronic kidney disease and end-stage renal disease	Annual
IDF Diabetes Atlas^14^	International Diabetes Federation	Multiple (e.g., Lancet Diabetes & Endocrinology); idf.org	Diabetes (global)	Periodic (approximately every 2–3 y)

National Institute of Neurological Disorders and Stroke (NINDS); National Heart, Lung, and Blood Institute (NHLBI); Centers for Disease Control and Prevention (CDC); National Center for Health Statistics (NCHS); National Institute of Diabetes and Digestive and Kidney Diseases (NIDDK); Centers for Medicare and Medicaid Services (CMS); International Diabetes Federation (IDF); United States Renal Data System (USRDS)

**Table 2:** Conventional Health Statistics Update Versus a Health Disparities Statistics Update.

Conventional statistical report	Proposed disparities update
Disease burden	Disease burden + distribution of burden
National averages	National averages + subgroup disparities
Mortality	Mortality + premature mortality
Incidence	Incidence + differential incidence
Prevalence	Prevalence + differential prevalence
Risk factors	Risk-factor disparities
Health-care utilization	Access and quality disparities
Costs	Distribution of economic burden
Single-year snapshot	Annual longitudinal trend
Disease-specific	Cross-disease
Epidemiologic description	Surveillance + accountability

**Table 3:** Structural template: mapping the annual American Heart Association (AHA) Heart Disease and Stroke Statistics Update to a potential annual neurologic health statistics update.

AHA Heart Disease and Stroke Statistics Update Feature^8,9^	Proposed Annual Neurologic Health Equity Statistics Equivalent
Continuous year-round data monitoring by a standing statistics committee, with literature review through the year prior to publication Sponsorship and co-production with NIH, CDC/NCHS, and other federal agencies Named chair, vice chair, and rotating volunteer writing group with mandatory conflict-of-interest disclosure Stable core chapters (mortality, prevalence, risk factors, cost, quality of care) preserved across editions for trend comparability Scope expanded over time as data matured (e.g., addition of a dedicated brain health chapter, expanded global data, beginning with the 2024 edition) Explicit framing of disparities and structural drivers (e.g., structural racism) as a core, expected element of the report, not a specialized supplement9 Publication in a high-impact specialty journal (Circulation) with a companion public-facing statistics dashboard and fact sheets	Standing organization secretariat (propose: SEQUINS, in partnership with ANA and NINDS Office of Disparities) that continuously tracks federal data releases rather than convening only at publication time Formal data-sharing and co-production relationships with NINDS, CDC/NCHS, AHRQ, CMS, and the US Census Bureau Named scientific steering committee with fixed term limits, published disclosure statements, and rotating condition-specific subcommittee leads Fixed core metrics and comparator strata held constant year over year, with methodologic changes flagged explicitly Rotating annual thematic chapter allowing scope to grow (e.g., pediatric neurology, long-term care) without disrupting the fixed core Race, ethnicity, sex, geography, and socioeconomic stratification embedded in every core table from the first edition, rather than issued as a one-time companion analysis Publication in a journal (propose: Equity Neuroscience) with a companion public dashboard and policymaker handout, as outlined in Dissemination and Translation

National Institute of Neurological Disorders and Stroke (NINDS); American Neurological Association (ANA); Centers for Disease Control and Prevention (CDC); National Center for Health Statistics (NCHS); Society for Equity Neuroscience (SEQUINS); Agency for Healthcare Research and Quality (AHRQ); Centers for Medicare and Medicaid Services (CMS)

**Table 4:** Proposed scope of Neurologic Equity Annual Report (NEAR).

Domain	Proposed Content	Comments
Core conditions	Stroke (ischemic, hemorrhagic) Alzheimer disease and related dementias Parkinson disease Epilepsy Migraine and other headache disorders Multiple sclerosis Amyotrophic lateral sclerosis	Additional Considerations: Traumatic brain injury Peripheral neuropathies Neuromuscular disorders Neuroinfectious diseases Neurodevelopmental disorders Sleep-related neurologic disorders
Core metrics	Incidence (New cases, age-adjusted incidence rates) Prevalence (Point and period prevalence) Mortality (Deaths, age-adjusted mortality, premature mortality) Years of life lost (YLLs) Disability (Years lived with disability -YLD, functional limitations) Disability-adjusted life-years (YLL + YLD) Risk factors (Vascular, metabolic, environmental, behavioral, social) Care access (Insurance, specialist access, travel, wait times) Quality of care (Evidence-based treatment, prevention, follow-up) Economic burden (Direct medical, indirect, caregiver costs) Geographic variation (State, county, urban/rural, region) Temporal trends (Annual and multiyear changes)	Key questions: Who is developing disease? Who is living with disease? Who is dying? Who is dying earlier than expected? Who is living with the greatest disability? Where is total disease burden greatest? Why are disparities occurring? Who can obtain neurologic care? Who receives high-quality care? Who bears the financial burden? Where are disparities concentrated? Are disparities widening or narrowing?
Comparator strata	Sex Race and ethnicity Census region, state, and rural/urban geography Socioeconomic position	Classifications: Non-Hispanic White, non-Hispanic Black, non-Hispanic Asian, Hispanic or Latino, non-Hispanic American Indian/Alaska Native, and other groups as sample size permits Income, insurance status, area-level deprivation indices, nativity, language
Classification standard	International Classification of Diseases, 10th Revision, Clinical Modification (ICD-10-CM)	Mapping: NIH Research, Condition, and Disease Categorization (RCDC) groupings for cross-report comparability
Trend analysis	Rolling 10-year trend lines per condition-by-stratum cell; annual and multiyear estimates of change	Reporting: Absolute and relative disparities with uncertainty intervals Use annual percent change, slopes, segmented/joinpoint, or multiyear regression approaches when appropriate Distinguish statistical from practical change and flag periods affected by case-definition or data-source changes.
Special features (rotating)	One in-depth thematic chapter per year supplementing the fixed statistical core	Examples: Rural neurologic workforce access; pediatric-to-adult transition disparities; language-access barriers

National Institutes of Health (NIH).

**Table 5:** Candidate data sources for the Neurologic Equity Annual Report (NEAR).

Source	Primary Contribution	Key Strength / Limitation
National Vital Statistics System (NVSS) and NCHS mortality files / CDC WONDER	Cause-specific mortality by demographic and geographic strata	Near-complete death ascertainment nationally, but death-certificate attribution and coding can miss neurologic contributions and vary by setting; race/ethnicity misclassification can bias subgroup estimates.
Healthcare Cost and Utilization Project Nationwide Inpatient Sample (AHRQ)	Hospitalization rates, in-hospital outcomes, cost of inpatient neurologic care	Large national discharge sample, but captures hospitalized events only Coding quality, admission thresholds, and differential access to hospital care can affect measured morbidity and disparities.
Medical Expenditure Panel Survey (MEPS)	Direct and out-of-pocket health care costs and utilization	Household-level cost detail; smaller sample limits precision for rarer conditions (e.g., ALS) in small strata
National Health and Nutrition Examination Survey (NHANES)	Disease and risk-factor prevalence with clinical/laboratory confirmation	Objective measurement (e.g., cognitive testing); modest sample size constrains rare-disease and small-subgroup estimates
National Health Interview Survey (NHIS)	Self-reported disease and risk-factor prevalence, functional status	Large annual sample supports demographic stratification; relies on self-report
National Ambulatory Medical Care Survey (NAMCS)	Outpatient visit patterns, specialist access	Captures ambulatory patterns, but sampling and coding limitations can constrain subgroup granularity Utilization reflects health-system contact and therefore may underestimate unmet need.
Behavioral Risk Factor Surveillance System (BRFSS)	State-level risk-factor and disease prevalence	Enables state and regional comparison; state-level methodologic variation affects cross-state comparability
US Census Bureau American Community Survey	Denominators and socioeconomic/demographic covariates	Strong capture in Medicare and dual-eligible populations, but race/ethnicity variables may be missing or misclassified and coverage is limited for younger commercially insured or uninsured adults.
CMS (Centers for Medicare & Medicaid Services) Medicare and Medicaid claims	Care patterns, cost, and outcomes in the population bearing the largest share of neurologic disease burden	Useful for cross-national benchmarking and DALYs, but estimates are modeled rather than directly measured and inherit assumptions about case definitions, inputs, and uncertainty.
Institute for Health Metrics and Evaluation (Global Burden of Disease) database	Modeled incidence, prevalence, and DALYs with US subnational estimates	Rich clinical detail but participating-site coverage and case ascertainment may not be nationally representative; access to specialty centers can itself be socially patterned.
Disease-specific registries (e.g., Paul Coverdell National Acute Stroke Program, National Alzheimer’s Coordinating Center, MS and ALS patient registries)	Condition-specific care-quality and natural-history detail	Near-real-time clinical detail, but nonrandom health-system participation, variable coding, and unequal access to participating systems limit national representativeness.
Oracle/Cerner real-world electronic health record data	Granular clinical and treatment-pattern data across health systems	Strong capture in Medicare and dual-eligible populations, but race/ethnicity variables may be missing or misclassified, and coverage is limited for younger commercially insured or uninsured adults.

Multiple Sclerosis (MS); Amyotrophic Lateral Sclerosis (ALS); Disability-Adjusted Life Year (DALY); World Stroke Organization (WSO)
