# Supplementary material for: Counting what counts: Time for an annual statistical update on neurologic health equity
Source: Equity Neurosci. Author manuscript; Available in PMC 2026 Oct 9. (PMC13645394; doi:10.1016/j.neuros.2026.100071)
Supplement: Supplementary file 1 [file NIHMS2211360-supplement-Supplementary_Tables_1-3.docx]

**SUPPLEMENTARY TABLES**

**Supplementary Table 1. S**trategic partners and proposed roles for Neurologic Equity Annual Report (NEAR).

| **Partner** | **Proposed Role** |
| --- | --- |
| Society for Equity Neuroscience | - Coordinating scientific secretariat; contributor recruitment; production of companion lay-facing and policymaker dissemination products - Editorial and professional-society sponsorship; dissemination to the multidisciplinary equity neuroscience community |
| American Neurological Association | - Co-sponsor process and consider doing a separate main statistical neurologic disease update; dissemination to the academic neurology workforce |
| NINDS Office of Global Health and Health Disparities | - Scientific co-sponsorship; liaison to federal data agencies; continuity with HEADWAY priorities and the NINDS health equity framework |
| Centers for Disease Control and Prevention (NCHS; Division for Heart Disease and Stroke Prevention) | - Mortality, hospitalization, and BRFSS data access; methodologic review of surveillance case definitions |
| Agency for Healthcare Research and Quality | - Access to and technical guidance on Nationwide Inpatient Sample and MEPS |
| Centers for Medicare & Medicaid Services | - Claims data use agreements for cost and care-pattern analyses in older and dual-eligible populations |
| US Census Bureau | - Population denominators and socioeconomic covariates; small-area estimation methodology |
| American Heart Association / American Stroke Association | - Methodologic consultation drawing on their statistics-update experience; potential co-publication for stroke-specific content |
| World Stroke Organization | - Methodologic consultation on global stroke burden estimation; alignment on stroke case definitions and comparator methodology |
| Condition-specific organizations (Alzheimer's Association, Epilepsy Foundation, National MS Society, ALS Association, American Migraine Foundation) | - Registry data access; patient and caregiver dissemination channels; thematic-chapter content review |
| Academic biostatistics and health-services research centers | - Independent analytic execution; methods transparency; trend-detection statistics |

United States (US); National Institute of Neurological Disorders and Stroke (NINDS); Amyotrophic lateral sclerosis (ALS); National Center for Health Statistics (NCHS); Medical Expenditure Panel Survey (MEPS); Behavioral Risk Factor Surveillance System (BRFSS)

**Supplementary Table 2.** Illustrative Annual Neurological Disparities Scorecard

| **Condition** | **Overall burden** | **Largest disparity** | **Trend** | **Major risk factor** | **Major care gap** | **Economic burden** |
| --- | --- | --- | --- | --- | --- | --- |
| Stroke | Very high | Race/geography | Upward | Hypertension | Prevention/treatment | High |
| Dementia | Very high | Race/sex | Upward | Age/vascular risks | Diagnosis/care | Very high |
| Parkinson disease | High | Geography/sex | Upward | Multiple | Specialty access | High |
| Epilepsy | High | SES/race | Variable | Multiple | Treatment access | High |
| Migraine | Very high | Sex/SES | Variable | Multiple | Diagnosis/treatment | High |
| Multiple sclerosis | Moderate | Sex/race/geography | Variable | Multiple | Specialty access | High |
| TBI | High | Race/geography/SES | Variable | Injury exposure | Rehabilitation | High |

Traumatic Brain Injury (TBI), Socioeconomic Status (SES)

**Supplementary Table 3.** Proposed Annual Publication Architecture

| **Output** | **Primary audience** | **Purpose** |
| --- | --- | --- |
| Full scientific report | Researchers/clinicians | Comprehensive evidence |
| Statistical supplement | Researchers | Reproducible data |
| Online dashboard | Public/policymakers | Interactive surveillance |
| Executive summary | Broad audience | Key findings |
| Infographics | Public/media | Communication |
| Disease-specific briefs | Specialty societies | Focused action |
| Data-gap report | Researchers/funders | Research priorities |
